# A genome-wide association study of a global rice panel reveals resistance in *Oryza sativa* to root-knot nematodes

**DOI:** 10.1093/jxb/erv470

**Published:** 2015-11-09

**Authors:** Stanley O. N. Dimkpa, Zobaida Lahari, Roshi Shrestha, Alex Douglas, Godelieve Gheysen, Adam H. Price

**Affiliations:** ^1^Institute of Biological and Environmental Sciences, University of Aberdeen, Aberdeen, AB243UU, UK; ^2^Department of Molecular Biotechnology, Ghent University (UGent), Coupure Links 653, B-9000, Ghent, Belgium; ^3^ Current address: Department of Crop and Soil Science, Rivers State University of Science and Technology, Nigeria

**Keywords:** GWAS, lectin, Mla, Nematode, resistance, rice diversity panel.

## Abstract

The root-knot nematode *Meloidogyne graminicola* is one of the most serious nematode pests worldwide and represents a major constraint on rice production. While variation in the susceptibility of Asian rice (*Oryza sativa*) exists, so far no strong and reliable resistance has been reported. Quantitative trait loci for partial resistance have been reported but no underlying genes have been tagged or cloned. Here, 332 accessions of the Rice Diversity Panel 1 were assessed for gall formation, revealing large variation across all subpopulations of rice and higher susceptibility in *temperate japonica* accessions. Accessions Khao Pahk Maw and LD 24 appeared to be resistant, which was confirmed in large pot experiments where no galls were observed. Detailed observations on these two accessions revealed no nematodes inside the roots 2 days after inoculation and very few females after 17 days (5 in Khao Pahk Maw and <1 in LD 24, in comparison with >100 in the susceptible controls). These two cultivars appear ideal donors for breeding root-knot nematode resistance. A genome-wide association study revealed 11 quantitative trait loci, two of which are close to epistatic loci detected in the Bala x Azucena population. The discussion highlights a small number of candidate genes worth exploring further, in particular many genes with lectin domains and genes on chromosome 11 with homology to the *Hordeum Mla* locus.

## Highlight

Assessing gall numbers in 331 cultivars of a rice diversity panel has identified two resistant landraces, 11 quantitative trait loci and good candidate genes for resistance to *Meloidogyne graminicola*.

## Introduction

The genus *Meloidogyne* (root-knot nematodes) has been identified as the most scientifically and economically important group of nematodes (Jones *et al.*, 2013). Within this genus, the rice root-knot nematode, *Meloidogyne graminicola* (Golden and Birchfield, 1965), is considered one of the most important pests of Asian rice (*Oryza sativa* L.) (Plowright and Bridge, 1990; De Waele and Elsen, 2007; Win *et al.*, 2011; Kyndt *et al.*, 2012; Ji *et al.*, 2013; Jones *et al.*, 2013).

*M. graminicola* is widely distributed, especially in South and Southeast Asia where it occurs in every rice-producing country surveyed so far (Jain *et al.*, 2012). It has been clearly shown to cause substantial damage to the rice root system (Plowright and Bridge, 1990). It can cause substantial yield losses to upland, lowland, and deepwater rice as well as in rice nurseries (Bridge *et al.*, 2005; Pokharel *et al.*, 2010; Win *et al.*, 2011). It is now considered one of the biotic causal agents of yield failure in tropical aerobic rice (Kreye *et al.*, 2009; De Waele *et al.*, 2013). Currently, *M. graminicola* infestation of rice-based agrosystems is only a serious problem in the tropics (Jones *et al.*, 2013) but the geographic areas affected might be expected to expand owing to climate change (Bebber *et al.*, 2013).

Second-stage juveniles (J2) invade rice roots just behind the root tips and migrate intercellularly towards the central cylinder. At this site, the J2 puncture selected vascular cells with their stylet and inject pharyngeal secretions, which ultimately leads to the reorganization of the plant cells into typical feeding structures called giant cells. These giant cells are imbedded in galls induced upon penetration of the roots by the J2. The J2 will start to swell and after three moults develops into a swollen female. For the remainder of its sedentary life, this female will feed on the giant cells (Gheysen and Mitchum, 2011). Although gall number is not always correlated with the level of ability of the plant to support nematode reproduction (susceptibility), it usually is a good indicator of the infection level (Dong *et al.*, 2007; Wang *et al.*, 2012). *M. graminicola* infection of rice plants may result in seedling mortality or reduced vigour, and a substantial reduction in vegetative and yield-contributing traits, yield, and seed quality (De Waele *et al.*, 2013; Patil and Gaur, 2014; Win *et al.*, in press). In an *M. graminicola*-infested rainfed lowland rice field in Bangledesh, and a similarly infested rainfed upland rice field in Thailand, nematicide application resulted in yield increases of 16–20% (or 1 t/ha) and 12–33%, respectively (Arayarungsarit, 1987; Sharma-Poudyal *et al.*, 2004).

The options to manage *M. graminicola* population densities below damage threshold levels are limited at the moment. Crop rotation, flooding, and the use of nematicides are practices that are often used to manage plant-parasitic nematodes in infested fields (Bridge *et al.*, 2005) but each of these practices has a number of important drawbacks (De Waele *et al.*, 2013). In this context, the identification and use of rice genotypes with resistance to *M. graminicola* offers an interesting alternative to limit the yield losses caused by this nematode species.

Accessions with good resistance to *M. graminicola*, showing consistently low nematode reproduction compared with susceptible reference genotypes, have been found in *O. longistaminata* A. Chev. & Roehrich and *O. glaberrima* Steud. (Plowright *et al.*, 1999; Soriano *et al.*, 1999; Cabasan *et al.*, 2012) but so far introgression of this resistance into Asian rice has not been very successful because the interspecific progenies do not express the same degree of resistance observed in African rice. In Asian rice, some resistance to *M. graminicola* has been reported (Jena and Rao, 1976; Yik and Birchfield, 1979; Sharma-Poudyal *et al.*, 2004; Prasad *et al.*, 2006); however, according to Bridge *et al.* (2005), only a few of these accessions are truly resistant and the majority are in fact susceptible to *M. graminicola* (Cabasan MTN, Lahari Z, Galeng J, Price AH, Gheysen G, and De Waele D. Re-examination of the resistance to *M. graminicola* infection within Asian rice genotypes. In preparation).

A comparative transcriptomics study of the site of *M. graminicola* infection (galls versus root tips) after 3 days in rice revealed 156 differentially expressed genes (Kyndt *et al.*, 2012). Key metabolic pathways, hormone homeostasis, and epigenetic processes are affected during *M. graminicola*-induced giant cell development in rice (Ji *et al.*, 2013).

Applying plant molecular genetics has resulted in the identification of several nematode resistance (R) genes or quantitative trait loci (QTLs) for resistance to sedentary endoparasitic nematodes, and some genes mapped to chromosomal locations or linkage maps (Messeguer *et al.*, 1991; Ganal *et al.*, 1995). A few have been cloned (Veremis and Roberts, 2000; Thurau *et al.*, 2010). One of the best characterized and commercially used root-knot nematode resistance genes is *Mi-1.2*, whch is found in the wild relative of tomato (*Lycopersicon peruvianum* complex) and confers resistance to several *Meloidogyne* species (Veremis and Roberts, 2000). In African rice, the major gene *Hsa-1* ^*Og*^ confers resistance to the cyst nematode *Heterodera sacchari* and has been mapped on chromosome 11 of an *O. sativa* x *O. glaberrima* interspecific cross (Lorieux *et al.*, 2003). In Asian rice, QTLs for partial resistance to *M. graminicola* were identified on chromosomes 1, 2, 6, 7, 9, and 11 using F_6_ recombinant inbred lines of a Bala x Azucena *O. sativa* mapping population (Shrestha *et al.*, 2007).

The variation in landraces provides an important reservoir of genetic diversity and potential sources of beneficial alleles for rice breeding and improvement (Zhang *et al.*, 2009). Thus germplasm collections are invaluable resources for screening more rice accessions for *M. graminicola* resistance and offer opportunities for plant breeders to apply conventional selection techniques if resistance is found, and even marker-assisted selection if the resistance can be mapped to the genome. Genome-wide association (GWA) studies with a global collection of 413 diverse rice (*O. sativa*) accessions including landraces and elite cultivars from 82 countries, known as the Rice Diversity Panel 1 (RDP1), have recently been conducted (Zhao *et al.*, 2011). The authors used 44100 single nucleotide polymorphisms (SNPs) (44K chip) as markers and systematically phenotyped 34 morphological, developmental, and agronomic traits over two consecutive field seasons. GWA analysis of the same RPD1 panel has been used to study the genetic architecture of rice aluminium tolerance (Famoso *et al.*, 2011) and QTLs for grain ionome composition (Norton *et al.*, 2014). In our study, we examined the root galling of 332 accessions of the RDP1 panel 2 weeks after inoculation with J2 of *M. graminicola* to evaluate the host response of these genotypes, to identify resistant genotypes, and to highlight potential QTLs and candidate genes worthy of further characterisation. Additional experiments with a small number of accessions were carried out to examine if observations were reproducible across differing experimental conditions (substrate, pot size, plant age, and laboratory).

## Materials and Methods

### *Host response of 332 rice accessions to* M. graminicola *infection 2 weeks after nematode inoculation*

This experiment was carried out in a growth chamber at the University of Aberdeen. The 332 cultivars screened were a subset of the 413 rice *O. sativa* accessions that have been genotyped with 44100 SNPs as previously described (Zhao *et al.*, 2011) (Supplementary Table 1). The 332 cultivars mostly belonged to one of five recognized subpopulations of rice, with 12 *aromatic*, 57 *aus*, 64 *indica*, 78 *tropical japonica*, and 88 *temperate japonica*, with the rest (32) an admix between those subpopulations. The seeds of the rice accessions were obtained from the National Rice Research Centre, USA, and bulked in Aberdeen, UK. The nematode screening method was adopted from Shrestha *et al.*, (2007). The accessions were assessed in batches of 40 using 10 temporally separated runs. Each run consisted of three plug trays (LBS, Colne, UK) of 84 wells each [36.5×36.5×50mm (length × width × height)] with each tray containing two replicates of 40 accessions from the RDP1 plus Azucena (*tropical japonica* rice variety) and Bala (*indica* rice variety) as reference genotypes. All genotypes were replicated six times, making a total of 252 plants per screening run. Within the three plug trays, a randomized complete block design was used where a block was one half of a tray. The trays were filled with sand and sown directly with two seeds per plug, which were then thinned to one seed per plug after 1 week. Every 2 weeks, another batch of 40 RDP1 accessions plus Azucena and Bala reference plants were sown in a separate screening run. J2 were collected from the roots of 50 mature rice plants as described in Shrestha *et al.* (2007), except that where cut galls from these stock plant roots were incubated for 7 days at 30°C in Shrestha *et al.*, (2007), we incubated them for only 3 days at 28°C.

The *M. graminicola* inoculum used was originally obtained from CABI (Egham, UK) and has been maintained on rice for 10 years in Aberdeen. The plants were inoculated with 200 J2 per plant 2 weeks after planting. Two weeks after inoculation, the roots were carefully removed and washed with tap water, and the nematode galls counted. An incubation period of 2 weeks was chosen to terminate the experiment near the end of one nematode life cycle to avoid secondary galling. The life cycle of *M. graminicola* is completed in 19 days at 22–29°C in well-drained soil (Pokharel *et al.*, 2010; Kyndt *et al.*, 2012; Ji *et al.*, 2013). The temperature in the growth chamber ranged from 28°C (day) to 25°C (night), with 50–70% humidity, and light of 350 µmol m^−2^ s^−1^ photosynthetically active radiation at plant height for 12h d^−1^. The plants were watered daily to field capacity and fertilized twice a week with Yoshida’s nutrient solution (Yoshida *et al.*, 1976). One of the runs included 30 randomly selected plants of RDP1 assessed for a second time to confirm the repeatability of the method.

### *Host response of 10 rice accessions to* M. graminicola *infection 5 weeks after nematode inoculation*

This experiment was carried out in a greenhouse, also at the University of Aberdeen. After the first screening with the 332 accessions, 10 rice accession were selected based on their root galling: five with the lowest nematode gall numbers (LD 24, Khao Pahk Maw, DV85, Gerdeh, and Ku115) and five with the highest gall numbers (Lusitano Zerawchanica, Karatalski, Taipei 309, Ta Mao Tsao, and Benllok) (Table 1). Seeds were sown in individual 13-cm diameter pots in the same sandy soil as used in the first experiment. Two weeks after planting, each seedling was inoculated with 100 J2 of *M. graminicola*. Each accession was replicated six times. The experiment was carried out in a greenhouse in July–September 2012 without supplementary light. The minimum temperature was 25°C. The plants were watered daily to field capacity and fertilized once a week with full Yoshida’s nutrient solution (Yoshida *et al.*, 1976). Gall numbers were counted 5 weeks after inoculation with J2.

**Table 1.** The 10 rice cultivars used in the 5-week inoculation experiment

Accession number	Accession name	Origin	Subpopulation
**298**	LD 24	Sri Lanka	*indica*
**330**	Khao Pahk Maw	Thailand	*aus*
**49**	DV85	Bangladesh	*aus*
**55**	Gerdeh	Iran	*aromatic*
**96**	KU115	Thailand	*tropical japonica*
**289**	Lusitano	Portugal	*temperate japonica*
**287**	Zerawchanica Karatalski	Poland	*temperate japonica*
**158**	Taipei 309	Taiwan	*temperate japonica*
**155**	Ta Mao Tsao	China	*temperate japonica*
**180**	Benllok	Peru	*temperate japonica*

### *Comparison of* M. graminicola *development on LD 24 and Khao Pahk Maw, with reference rice genotypes*

These experiments were carried out at Ghent University. The *M. graminicola* culture was provided by Prof. Dirk De Waele (University of Leuven, Belgium) and was originally isolated from rice in the Philippines. *M. graminicola* was maintained on *O. sativa* cv. Nipponbare in potting soil or on the grass *Echinochloa crusgalli*. Three-month-old infected plants were used to extract J2 of *M. graminicola* using a modified Baermann method (Luc *et al.*, 2005). Seventeen-day-old rice seedlings in SAP medium (Reversat *et al.*, 1999) were inoculated with ±200 freshly harvested J2 per plant as previously described (Nahar *et al.*, 2011). Eight plants were analysed per genotype. Rice (*O. sativa*) genotypes CO39 (ssp. *indica*) and Taichung65 (ssp. *japonica*) were used as susceptible references and TOG5674 (*O. glaberrima* from the International Rice Research Institute) as a resistant reference genotype (Cabasan *et al.*, 2012). The infection level of the plants and the developmental stages of the nematodes were evaluated 2 days after inoculation (DAI) and 17 DAI using acid fuchsin staining (Nahar *et al.*, 2011). The root and shoot lengths were also recorded.

### Statistical analyses

**Fig. 1.**
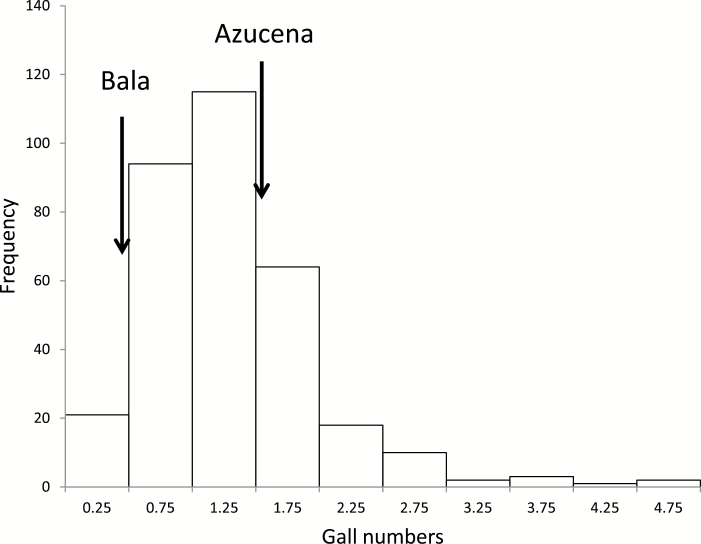
Frequency distribution of gall numbers in 332 RDP1 accessions relative to the check genotypes Azucena and Bala.

Collected experimental data of the RDP1 screen were corrected for block effects (within each run) using Minitab 16 (Minitab Inc, USA). To account for differences between runs, the mean gall numbers of the six replicates of each accession in each run was divided by the combined mean of the check genotypes Azucena and Bala in that particular screening. Because the relative gall number across all RDP1 accessions tested was not normally distributed (Fig. 1), it was square root transformed before further analysis. One-way ANOVA was carried out using Minitab 16 to compare relative galls numbers between rice subpopulations as given in Zhao *et al.* (2011). Pairwise comparisons among subpopulation means were achieved with Tukey’s method at a 5% level of significance. Correlation analyses using Minitab were conducted between relative means of nematode gall numbers from 30 accessions that were included in two separate runs (to assess reproducibility between runs), and between the relative means of gall number from the 10 accessions assessed in the 332-cultivar screen and the absolute number of galls in the 5-week inoculation experiment. In the analysis of nematode development, ANOVA was performed using the software SPSS (Version: SPSS Statistics 22). The means of the control and treated groups were compared by Duncan’s multiple mean comparison test at the 5% level of significance.

GWA analysis was performed on all the 332 accessions and also for each of the four subpopulations *indica*, *aus*, *tropical japonica*, and *temperate japonica* according to Zhao *et al.* (2011). Briefly, a linear mixed effects model (LMM) was used to model the association between each SNP and the phenotype (mean relative gall number) whilst accounting for population structure and potential relatedness between accessions using efficient mixed model analysis (EMMA) (Yu *et al.*, 2006). For the fixed effects, population structure (Price *et al.*, 2006) was included as the first four principal components of a principal components analysis of all SNPs across accessions (Zhao *et al.*, 2010). Relatedness was incorporated into models as a random effect, estimated using a kinship matrix (Zhao *et al.*, 2011) that measured the genetic similarity between individuals as the proportion of times a given pair of accessions had the same genotype across all SNPs (IBS values). For models of each of the four subpopulations, only random effects were incorporated.

An association between a SNP and phenotype was considered significant if the minor allele frequency (MAF) was >5% and the *P*-value from LMM <1×10^–4^. This threshold has been used previously based on the upper-limit false discovery rate, determined from the candidate genes in the same approach as used previously (Li *et al.*, 2010; Zhao *et al.*, 2010; Famoso *et al.*, 2011; Zhao *et al.*, 2011; Norton *et al.*, 2014). For each SNP considered significant with this method, we determined if they were also significant after applying a Benjamini–Hochberg correction. All reporting of SNP position used Pseudomolecules version 7 from the Rice Genome Annotation Project (RGAP) (http://rice.plantbiology.msu.edu/index.shtml).

### Candidate gene selection

In this study, QTLs were considered to be within 200kb of a significant SNP. The 200-kb window was selected to fall within the estimated window of linkage disequilibrium (LD) decay in rice (~50–500kb) (Mather *et al.*, 2007; Rakshit *et al.*, 2007; McNally *et al.*, 2009). This is close to the average for different subpopulations in this panel (100kb for *indica*, 200kb for *aus* and *temperate japonica*, and 300kb for *tropical japonica*) identified by Zhao *et al.* (2011) and is the approach for listing candidate genes used for this population by those authors. If more than one significant SNP was located within 200kb of another, they were considered to be one QTL. Genes within 200kb of the chosen significant SNP were selected using rice Pseudomolecule version 7. All genes described as transposon-related or retrotransposon-related were not selected. To identify which of these genes are good candidates for resistance to nematodes, we examined further information from the RGAP, such as gene ontology classification, PFAM hits, and Interpro hits, which provide insight into gene function and known gene orthology and homology.

## Results

### *Host response of 332 rice accessions to* M. graminicola *infection 2 weeks after nematode inoculation*

**Fig. 2.**
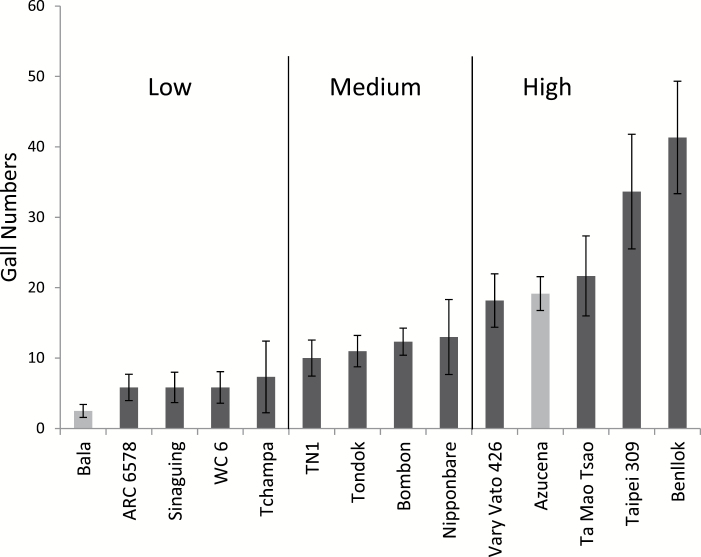
Plot of example accessions in one run, including reference cultivars Azucena and Bala and 12 RDP1 accessions grouped as four low, four medium, and four high. Bar is standard error.

Figure 1 shows the frequency distribution of the relative gall numbers, while all values are given in Supplementary Table 1. As an example, the results for some accessions of the RDP1 in one run that show low, medium, and high galls numbers are presented in Fig. 2. Within most runs there was highly significant variation due to genotype (significant in 9 out of 10 runs), indicating genetic variation for gall numbers is readily detectable. The result of the repeat run with the 30 randomly selected rice accessions revealed a strong correlation with the initial assessment (r = 0.730, *P* < 0.001) (data not shown), indicating the method is highly repeatable.

**Fig. 3.**
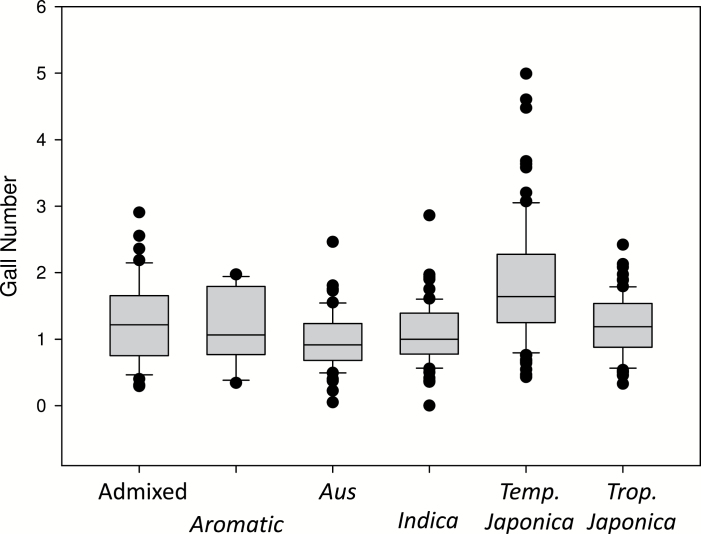
Box plot of relative gall numbers in the different subgroups of rice. *Temperate japonicas* were significantly higher.

The mean number of galls relative to Azucena and Bala was 1.3 while the highest values obtained were over four times the average of the reference genotypes. The three highest scoring accessions were all temperate *japonicas*: Ta Mao Tsao from China (4.6 relative galls), Taipei 309 from Taiwan (4.5), and Benllok from Peru (5). LD 24, an *indica* accession from Sri Lanka, showed no galls in any of the six replicates whereas another accession from Thailand, Khao Pahk Maw from the *aus* subpopulation, had just one gall in one of the replicates with zero galls in the other five replicates. There was a significant difference (*P* < 0.001) in gall number between rice subpopulations that explained 21% of the variation in relative gall number (Fig. 3). The *temperate japonica* had the highest number of galls, followed by tropical *japonica*, *aromatic*, *indica*, and *aus*. A Tukey’s test indicated that the number of galls in the *temperate japonicas* was significantly higher than in the other subpopulations.

### *Host response of 10 rice accessions to* M. graminicola *infection 5 weeks after nematode inoculation*

**Fig. 4.**
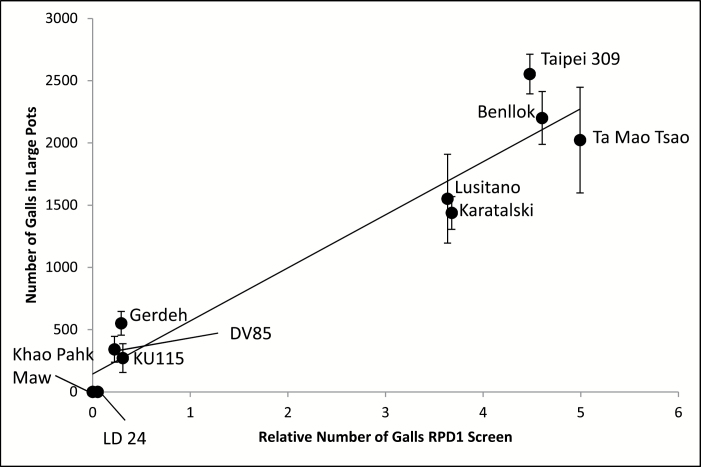
Plot of mean and standard error of gall numbers in 10 RDP1 accessions inoculated for 5 weeks in large pots versus the relative gall numbers obtained after 2 weeks when RDP1 was screened. Note no galls were detected in Khao Pahk Maw and LD 24. Correlation r = 0.967.

The number of galls observed in the 10 selected accessions of the RDP1 5 weeks after inoculation was highly significantly different between accessions (*P* < 0.001, R^2^ = 79%) and correlated very strongly (r = 0.970, *P* < 0.001) with the nematode gall numbers in the initial 2-week nematode screening of 332 diverse cultivars (Fig. 4). The *indica* LD 24 and *aus* Khao Pahk Maw had zero galls in the six replicates of this experiment, confirming the same result in the initial 2-week nematode screening of the RDP1.

### *Comparison of* M. graminicola *development on LD 24 and Khao Pahk Maw with that on reference rice genotypes*

**Fig. 5.**
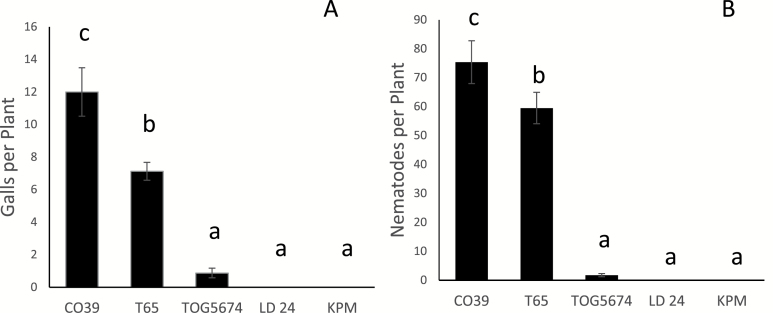
Responses of rice genotypes to root knot nematode *M. graminicola* 2 DAI. Two susceptible genotypes, CO39 and T65, and one resistant genotype, TOG5674, were used as control treatments to analyse the susceptibility of the rice accessions LD 24 and Khao Pahk Maw. Each treatment was a pool of eight individual plants. The number of inoculated nematodes per plant was ±200 J2. The response of each genotype was evaluated in terms of **(A)** number of galls per plant and **(B)** number of nematodes (J2) per plant. Each bar with standard error (±SE) represents the average number of galls or nematodes. Letter(s) on error bars are based on statistical analysis (Duncan’s multiple range tests after one-way ANOVA). Genotypes having different letters are statistically significant (*P* = 0.05). The experiment was done twice with similar results.

Two *M. graminicola*-susceptible *O. sativa* genotypes, CO39 and Taichung65 (T65), and one *M. graminicola*-resistant African rice, *O. glaberrima,* genotype TOG5674 were used as controls. LD 24 and Khao Pahk Maw showed nematode infection-resistance comparable to that of TOG5674. At 2 DAI, on average less than two J2 of *M. graminicola* had penetrated the TOG5674 roots and no J2 had penetrated the roots of LD 24 and Khao Pahk Maw (Fig. 5 A, B). The susceptible rice genotypes CO39 and T65 had 7–12 galls per plant, with 59–75 J2 in their roots (Figs 5 A, B).

**Fig. 6.**
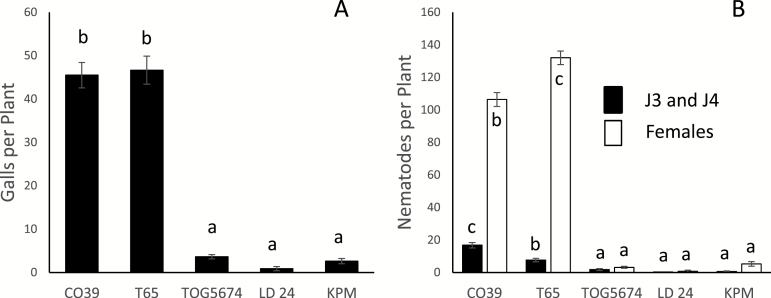
Responses of susceptible CO39 and T65, and resistant TOG5674, LD 24, and Khao Pahk Maw rice genotypes to *M. graminicola* 17 DAI with ±200 nematodes. Response of each genotype is evaluated in terms of **(A)** number of galls per plant, **(B)** number of nematodes (J3 and J4, and females) per plant. Each bar with standard error (±SE) represents the average number of galls or nematodes recorded on eight plants for each genotype. Letter(s) on error bars are based on statistical analysis (Duncan’s multiple range tests after one-way ANOVA). Genotypes having different letters are statistically significant (*P* = 0.05). The experiment was done twice with similar results.

At 17 DAI, the trend of nematode-infection level in resistant and susceptible rice genotypes was similar to that at 2 DAI. The number of galls and developed females were comparable in the resistant rice genotype TOG5674 and Khao Pahk Maw (about five females per plant) and this was significantly lower than in the susceptible rice genotypes, which had on average >100 females per plant (Fig. 6). Most remarkably, in LD 24 we observed on average less than one gall and one female per plant (Fig. 6).

### GWA study

**Fig. 7.**
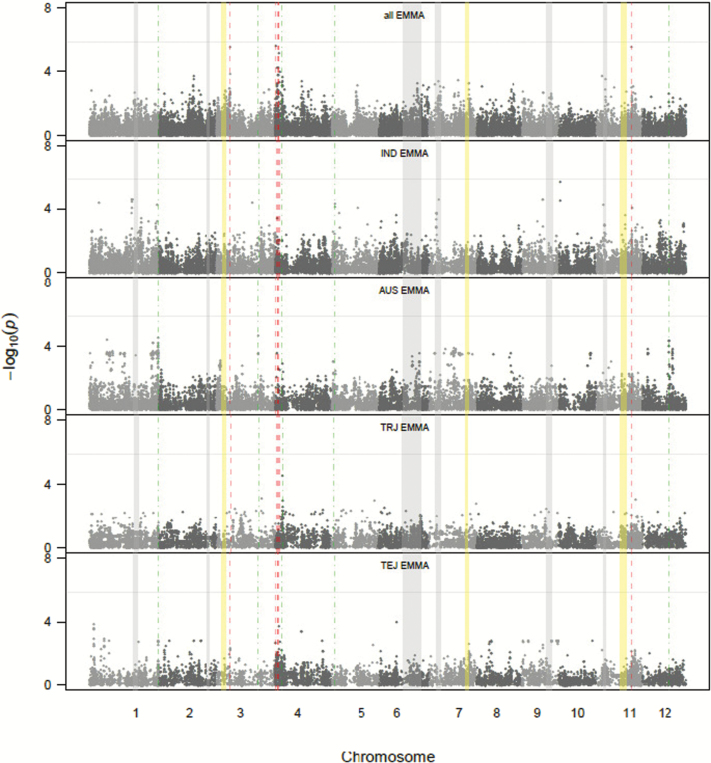
GWA of nematode gall numbers across and within subpopulations. *P*-values from the mixed model for all cultivars in top panel (all), and for *tropical japonica* (TRJ), *temperate japonica* (TEJ), *indica* (IND), and *aus* (AUS) subpopulations individually in subsequent panels. The aromatic subpopulation was not included owing to its small sample size. *X*-axis indicates the SNP location along the 12 chromosomes *y*-axis is the −log 10 (*P* value). Red dotted lines indicate where the associated SNPs in the ‘all’ analysis were detected while green were detected in one of the subpopulation analyses. Grey shading indicates QTLs detected in [33] while those in yellow are biallelic epistatic interactions also detected in the [33] study but not reported.

The result of the GWA scans is summarized in Fig. 7 and Table 2. The qq plots are presented in Supplementary Fig. 1. A total of 18 significant SNPs were associated with nematode gall numbers using the EMMA, judged to represent 11 QTLs (Table 2). Five of the SNPs were not significant after a Benjamini–Hochberg correction, which represents four of the 11 QTLs. This gives stronger confidence for the seven QTLs that were detected after this correction, but it does not mean the others are not true QTLs. When analysing the population as a whole, we identified eight SNPs as associated with gall number, appearing to represent five QTLs, on chromosomes 3, 4 (three QTLs), and 11. These are identified with red dotted lines in Fig. 7. In the separate subpopulation analysis, we detected seven significant SNPs representing three QTLs on chromosome 1, 4, and 12 in the *aus* subpopulation, two SNPs from one QTL on chromosome 5 in *indica*, and one SNP on chromosome 4 in the *tropical japonica*. No SNP was observed in *temperate japonica*.

**Table 2.** Eighteen SNPs with significance association with relative nematode gall numbers using EMMA. SNPs in bold were significant after Benjamini–Hochberg correction

Suspected QTLs	SNP id	Analysis	Chromosome	Position (bp)	*P*-value	MAF (%)
1	**dd1002102**	**AUS**	**1**	**42554085**	**2.81E−05**	**8.6**
1	**id1027818**	**AUS**	**1**	**42581030**	**7.57E−05**	**5.6**
1	**id1027825**	**AUS**	**1**	**42582856**	**6.37E−05**	**5.5**
3.1	**id3004493**	**All**	**3**	**8544211**	**2.75E−06**	**45**
3.1	**id3004510**	**All**	**3**	**8573136**	**3.28E−06**	**45**
3.1	**id3004522**	**All**	**3**	**8595237**	**3.28E−06**	**45**
3.2	**id3011085**	**AUS**	**3**	**8596299**	**2.18E−05**	**5.4**
4.1	**id4000274**	**All**	**4**	**474253**	**2.27E−06**	**8.6**
4.2	id4000769	All	4	1405412	6.59E−05	8.4
4.3	id4000913	All	4	1876908	6.81E−05	12
4.4	**id4001092**	**All**	**4**	**2457675**	**8.00E−06**	**17**
4.5	id4001733	TRJ	4	4276025	3.06E−05	14
5	id5001000	IND	5	1572494	7.78E−05	7.7
5	id5001019	IND	5	1656600	5.18E−05	6.8
11	**id11008353**	**All**	**11**	**22245292**	**2.65E−06**	**5.2**
12	**id12005724**	**AUS**	**12**	**16909682**	**9.95E−05**	**5**
12	**id12005764**	**AUS**	**12**	**17074099**	**4.50E−05**	**11**
12	**id12005794**	**AUS**	**12**	**17282922**	**4.51E−05**	**6.8**

None of the QTLs detected by the GWA study are co-located with main effect QTLs reported for the same trait in the Bala x Azucena mapping population (Shrestha *et al.*, 2007) (grey shading in Fig. 7). However, Shrestha *et al.* (2007) did not report biallelic epistatic interactions. Subsequent analysis of that data using the programme QTLMapper version 1 (Wang *et al.*, 1999a; Wang *et al.*, 1999b) revealed two epistatic interactions in the Bala x Azucena mapping population, both involving a locus on chromosome 11 at around 13–18 Mbp, one with a locus on chromosome 3 at around 4 Mbp (explaining 23% of the genetic variation) and another with a locus on chromosome 7 around 24 Mbp (explaining 18% of the genetic variation) (yellow shading in Fig. 7). These two epistatic interactions were confirmed to be both strong and highly significant by a three-way ANOVA of the gall numbers in the 4-week experiment reported in Shrestha *et al.* (2007) with markers RG409, RG650, and a1245y as factors. This model explains 30% of the variation for gall numbers, and significant interactions were detected between RG409 and a1245y (*P* = 0.001) and between RG650 and a1245y (*P* < 0.001). These interactions are presented graphically in Supplementary Fig. 2. The epistatic loci on chromosomes 3 and 11 are within a few megabase pairs of QTLs detected by the GWA study and may well be within expected confidence intervals of biallelic linkage mapping.

A total of 493 positional candidate genes were selected within 200kb of the identified significant SNPs/genomic regions representing 11 suspected QTLs for nematode resistance (see Supplementary Table 2). These included genes without functional annotation (191 expressed proteins and 34 hypothetical proteins). Of these 493 genes, 16 were previously identified as differentially regulated after 3 or 7 days in rice roots infected with *M. graminicola* (Kyndt *et al.*, 2012) (15 up-regulated and one down-regulated) whereas 51 were identified as up-regulated and 6 down-regulated in the giant cells of *M. graminicola*-infected rice by Ji *et al.* (2013) (indicated in Supplementary Table 2). Five genes were differentially regulated in both studies. These genes may be good candidate genes for nematode resistance.

## Discussion

### *Host response of the rice cultivars examined to* M. graminicola *infection*

The present study screened 332 diverse rice accessions from the RDP1 for gall development of *M. graminicola*. We found significant variation in the degree of susceptibility of *O. sativa* accessions to infection. Thirty randomly selected rice accessions from the RDP1 were used to repeat the 2-week experiment, confirming the repeatability of the method, while the method was further evaluated using a longer pot experiment for 5 weeks after inoculation using 10 selected accessions of the RDP1. Our results provide robust evidence that this is a good method to screen for rice–nematode interactions. The mean gall number ranged from 0 galls per plant in cultivar LD 24 (in a run with a mean number of galls in Azucena and Bala of 14.2 and 1.3 respectively) to 41 galls per plant in Benllok (in a run with a mean number of galls in Azucena and Bala of 19.2 and 2.5 respectively) when assessed 2 weeks after inoculation. In the larger, longer pot experiments, Benllok had over 2000 galls whereas LD 24 still had 0 galls per plant (Fig. 4). The correlation coefficient reflected a strong significant positive relationship between gall numbers per accession in the two experiments (2 and 5 weeks after inoculation). The differential susceptibility of *O. sativa* accessions to *M. graminicola* revealed by the results of our experiment supports the findings reported by other authors (Plowright *et al.*, 1999; Soriano *et al.*; 1999, Shrestha *et al.*, 2007) that accessions differ in their reaction to *M. graminicola*. However, the remarkable result of the present data is that they suggest for the first time a complete resistance to infection of *M. graminicola* of two *O. sativa* accessions, LD 24 (an *indica* from Sri Lanka) and Khao Pahk Maw (an *aus* from Thailand). These accessions had no nematode galls in the six replicates of the 5-week experiment (Fig. 4).

This resistance of LD 24 and Khao Pahk Maw was confirmed by an independent study on the same cultivars in a different laboratory using a different nematode population. Analysis of the infected rice plants at 2 DAI revealed the absence of penetrated nematodes in the resistant accessions, suggesting that the resistance acts at a very early step during the infection by preventing or delaying penetration. This indicates a different resistance mechanism than in the case of *Mi-1.2*, where the nematodes enter the roots but induce a rapid necrosis when attempting to induce giant cells (Williamson and Hussey, 1996). Analysis of LD 24 and Khao Pahk Maw roots at 17 DAI showed that very few nematodes had entered the root later and developed into females.

The very low nematode infection was not due to poor root growth because both Khao Pahk Maw and LD 24 had more roots than the susceptible genotypes CO39 and T65 (data not shown). Nevertheless, it is important to know how LD 24 and Khao Pahk Maw perform in the field and how nematode reproduction in these accessions compares to that in susceptible accessions. Experiments in raised beds in the Philippines have already confirmed the resistance in field-like conditions (Cabasan *et al*., in preparation).

The fact that reports of resistance to root-knot nematodes in *O. sativa* are rare and irreproducible (Cabasan *et al*., in preparation) was a major motivation to further analyse the accessions that showed no galls in the RDP1 screening. Shrestha *et al.* (2007) reported QTLs for partial resistance to *M. graminicola* in *O. sativa* and resistance in the *O. glaberrima* accession CG14. Lorieux *et al.* (2003) reported resistance to *Meloidogyne* spp. and the cyst nematode *Heterodera sacchari* in varieties of the African rice cultivated species *O. glaberrima*. Effort has been made in the past to transfer the *Hsa-1* ^*Og*^ gene from rice *O. glaberrima* chromosome 11 to *O. sativa* through interspecific crossing but the interspecific progenies tested so far have not been able to express the same degree of resistance (Plowright *et al.*, 1999; Lorieux *et al.*, 2003).

Phenotyping of the diversity panel has provided valuable information about the range and distribution of nematode susceptibility in *O. sativa* and offered new insights into the evolution of the susceptibility of the rice subspecies to nematode establishment (i.e. ability to form galls). The mean number of nematode galls in the subspecies *japonica* was greater than 1.5 times that of *indica* (*P* < 0.05) and the relative number of nematode galls in the five subpopulations indicates that *temperate japonicas* had most galls on average (*temperate japonica* > *tropical japonica* ≈ *aromatic* ≈ *indica* ≈ *aus*), which may reflect the fact that the nematode is not a pest of temperate rice production so no selection has been operating to introduce resistance. It has been suggested that the range of most crop pests and pathogens are moving in a poleward direction under the influence of global warming at an average rate of 2.7 km per year, with wide variation between types and individual pests/pathogens (Bebber *et al.*, 2013). It may therefore be expected that the root-knot nematode will become an increasingly important pest in temperate rice regions as the planet warms. The high gall numbers observed in the *temperate japonicas* should be a cause of concern in this context.

### Genetic loci affecting galling

In the interest of brevity, we are highlighting only two of the most noteworthy functional links between genes under QTLs and nematode resistance. First it is noteworthy that six of the identified 11 QTL regions contain genes annotated as containing lectin domains (See Supplementary Table 2). There is one gene in QTL 1 (annotated as S-locus lectin protein kinase), one in QTL 3 (annotated as lectin-like receptor kinase), two in QTL 4.1 (both legume lectins beta domain containing protein), three in QTL 4.2 (one annotated as above and two jacalin-like lectin domain containing protein), two in QTL 4.4 (both the legume lectin type), and one in QTL 5 (lectin protein kinase). These 10 genes represent only 8% of those genes in rice annotated with ‘lectin’ because there are 125 of them. But given that these 11 regions together are only 0.55% of the genome [(0.2×11)/400 Mb], this is more ‘lectin’ genes than chance would suggest. Doing similar analysis for genes with common annotations reveals that these lists contain 3.75% of the 160 ‘peroxidase’ genes, 2.6% of the 380 ‘transferase’ genes, 1.5% of the 470 ‘synthase’ genes, 1.0% of the 192 ‘ubiquitin’ genes, and 0% of the 221 ‘reductase’ genes. Also noteworthy here is that in the QTL 4.1, three additional genes have a lectin legB domain despite not being annotated as lectin genes (LOC_04g01860, 01874, and 01890). Lectins are recognized as proteins associated with plant resistance to nematodes and transgenic alteration of lectin expression has been shown to confer nematode resistance (Fuller *et al.*, 2008).

The second observation worth highlighting is that QTL 11.1 contains clusters of both *mla1*-like genes and highly homologous stripe rust-resistance proteins. In the gene list are seven stripe rust-resistance proteins surrounding two genes annotated as *mla* or *mla1* putative. *Mla* is a powdery mildew resistance gene of barley (*Hordeum vulgare* L.) (Wei *et al.*, 1999). It has very strong homology to stripe rust-resistance genes [e.g. 64% amino acid identity between LOC_Os11g37790 (mla putative) and LOC_Os11g37774 (rust stripe resistance protein Yr10)]. Two of these stripe rust genes were shown to be down-regulated in giant cells (LOC_Os11g37774 and LOC_Os11g37860) (Ji *et al.*, 2013) while one (LOC_Os11g37850) is up-regulated in infected rice roots after 7 days (Kyndt *et al.*, 2012). Because this locus may co-localize with the epistatic QTL identified in the Bala x Azucena mapping population, it is relevant to examine the Bala and Azucena genome sequences (Fastq data have been deposited in the NCBI Short Read Archive Acc_ID SRA050654.1). This reveals that the stripe rust genes LOC_Os11g37759, Os11g37774, and Os11g37780 appear to be missing in Bala while the two *mla1* genes are missing in both Azucena and Bala, suggestive of major chromosomal re-arrangement in this region. Seifi *et al.* (2011) have provided strong genetic evidence in tomato that the nematode- and insect-resistance gene *Mi-1* co-segregates or is causally related to mildew resistance.

The two cultivars LD 24 and Khao Pahk Maw are remarkable because they hardly form galls compared to all the other accessions assessed. Because this phenotype is very rare (in the RDP1 and across *O. sativa*), GWA studies will not detect it (because alleles with frequency below 5% will not be considered). If these two accessions are resistant because of the presence of the same single and very rare gene (an assumption that might well not be true) then it should be possible to identify a likely position of that gene based on the SNP data. An attempt was made to do that in three steps. First, SNPs were filtered to remove all SNPs that were not common between LD 24 and Khao Pahk Maw, leaving about 20 000 SNPs. Second, SNPs were eliminated if they were found in the nearest accession to LD 24 based on principal components analysis, the Indonesian *indica* Seratoes Hari that had a mean gall number value of 1 (i.e. exactly on the mean of Bala and Azucena). This left 3737 SNPs. Finally, because the region in LD 24 and Khao Pahk Maw must be rare, all SNPs within this group with a MAF value below 5% were identified. There were 16 SNPs where the allele in the resistant cultivars had a low frequency. Six of these fall in a cluster between 42.31 and 42.60 Mbp on chromosome 1, five cluster between 0.95 and 1.24 Mbp on chromosome 3, and three cluster between 26.32 and 26.33 Mbp on chromosome 11, leaving two singletons at 2.47 and 18.45 Mbp on chromosome 11. Further mapping will be required to confirm if these loci really harbour resistance genes but it is noteworthy that at 26.4Mb on chromosome 11 is a resistance gene homolog (LOC_Os11g43700) annotated as RGH1A, which is associated with the *Mla* locus in barley (Wei *et al.*, 2002).

In conclusion, we have demonstrated significant variation in the degree of susceptibility of *O. sativa* accessions to *M. graminicola* infection. For the first time, two *O. sativa* accessions (LD 24 and Khao Pahk Maw) have been revealed to have complete resistance to infection by *M. graminicola*. Significant differences were seen in the number of galls among the rice subpopulations. The *temperate japonicas* had a higher number of galls on average than the tropical *japonicas,* aromatics*, indicas*, and *aus*. We have also identified candidate genes worth pursuing for nematode resistance.

## Supplementary data

Supplementary data are available at *JXB* online.

Supplementary Fig. 1 qq plots for all data with naïve and mixed model results, plus naïve for each of the four subpopulations. Dotted red line is the one-to-one line.

Supplementary Fig. 2 Epistatic interaction for gall number in the Bala x Azucena mapping population. The mean number of galls 4 weeks after inoculation with *M. graminicola* for all six combinations of allelic combinations of markers in order of RG409 (chromosome 3), RG650 (chromosome 7), and a1245y (chromosome 11) where A is Azucena and B is Bala. Bar is standard error. Bars that do not share the same letter above are different by Tukey’s test at *P* < 0.05. Note this is a further analysis of data first reported in Shrestha *et al.* (2007). A three-way ANOVA with these markers as factors explains 30% of the variation for gall numbers, and significant interactions are detected between RG409 and a1245y (*P* = 0.001) and between RG650 and a1245y (*P* < 0.001).

Supplementary Table 1 Accession details and relative gall numbers for the Rice Diversity Panel 1.

Supplementary Table 2 List of Gene 200 kb either side of the significant SNPs.

Supplementary Data
