# Supplementary material for: A genome-wide association study of a global rice panel reveals resistance in Oryza sativa to root-knot nematodes
Source: J Exp Bot. 2015 Nov 9;67(4):1191–200. doi: 10.1093/jxb/erv470 (PMC4753847; doi:10.1093/jxb/erv470)
Supplement: Supplementary Data [file supp_67_4_1191__index.html]

A genome-wide association study of a global rice panel reveals resistance in Oryza sativa to root-knot nematodes — A genome-wide association study of a global rice panel reveals resistance in Oryza sativa to root-knot nematodes — Supplementary Data 

# A genome-wide association study of a global rice panel reveals resistance in *Oryza sativa* to root-knot nematodes

## Supplementary Data

Data files

- Supplementary\_figures.pdf - Supplementary Data
- Supplementary\_tables.xlsx - Supplementary Data
