# Supplementary material for: A genome-wide association study of a global rice panel reveals resistance in Oryza sativa to root-knot nematodes
Source: J Exp Bot. 2015 Nov 9;67(4):1191–200. doi: 10.1093/jxb/erv470 (PMC4753847; doi:10.1093/jxb/erv470)
Supplement: Supplementary Data [file supp_erv470_Supplementary_figures.pdf]

A genome-wide association study of a global rice panel reveals resistance in Oryza sativa to root-knot nematodes

Stanley O N Dimkpa, Zobaida Lahari, Roshi Shrestha, Alex Douglas, Godelieve Gheysen, and Adam H Price

Supplemental File

Supplementary Figure 1 qq plots for all data with naïve and mixed model results, plus naïve for each of the four subpopulations. Dotted red line is the one to one line.

Supplementary Figure 2 Epistatic interaction for gall number in the Bala x Azucena mapping population. The mean number of galls four weeks after inoculation with M. graminicola for all six combinations of allelic combinations of markers in order of RG409 (chromosome 3), RG650 (chromosome 7) and a1245y (chromosome 11) where A is Azucena and B is Bala. Bar is standard error. Bars that do not share the same letter above are different by Tukey's test at $P < 0.05$. Note this a further analysis of data first reported in Shrestha et al., (2007). A three way ANOVA with these markers as factors explains 30% of the variation for gall numbers, and significant interactions are detected between RG409 and a1245y ($P = 0.001$) and between RG650 and a1245y ($P < 0.001$).

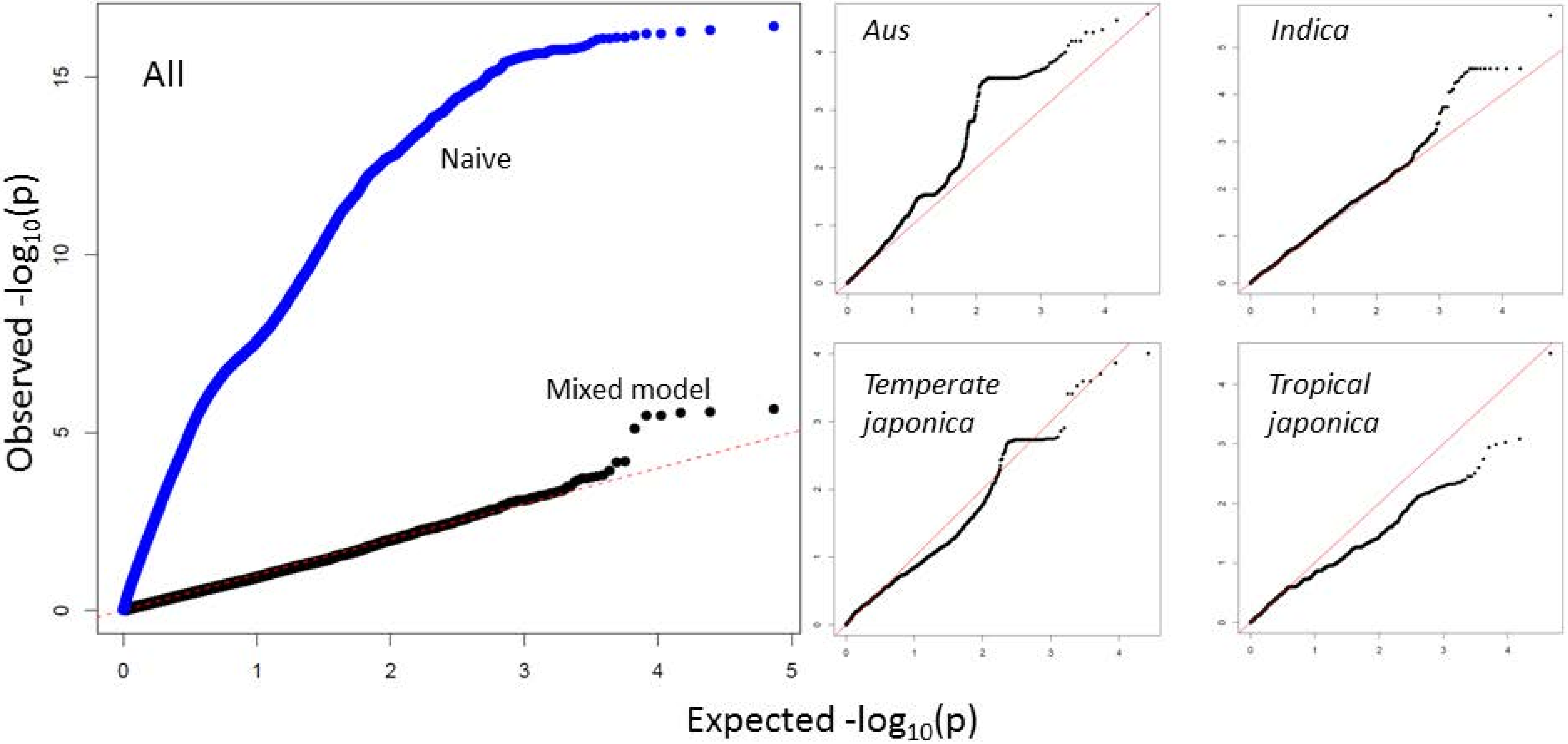

Supplementary Figure 1 qq plots for all data with naïve and mixed model results, plus naïve for each of the four subpopulations. Dotted red line is the one to one line.

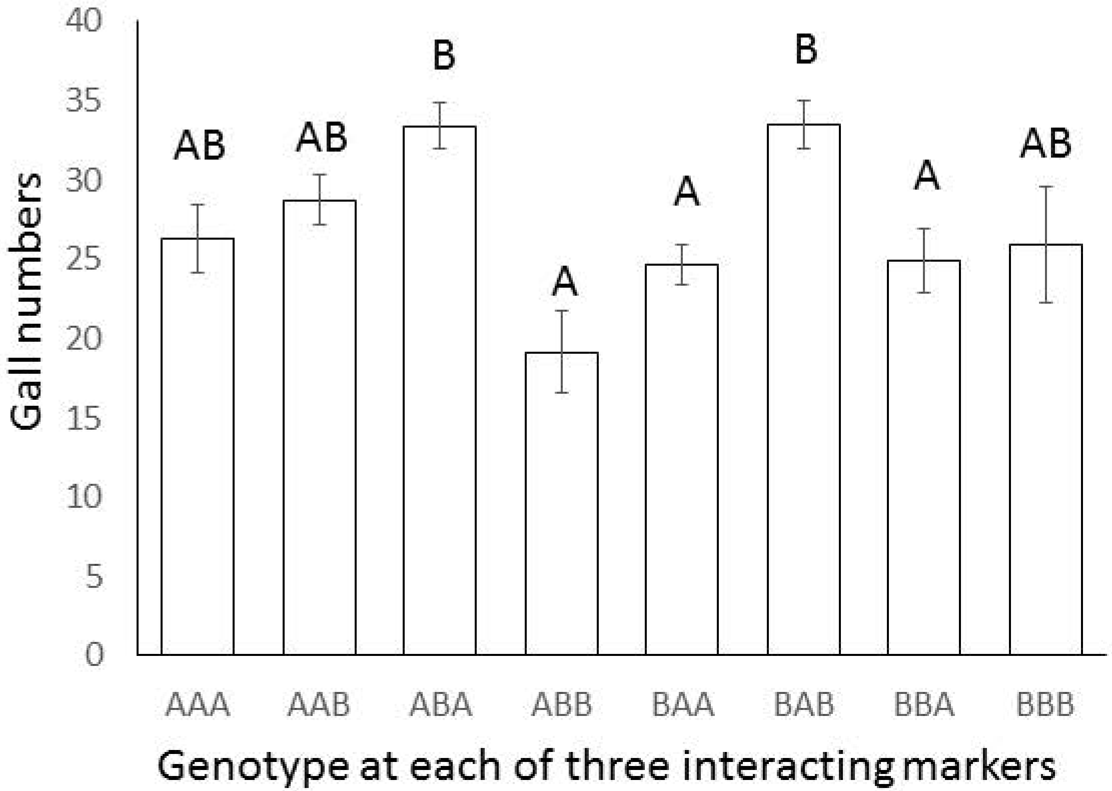

Supplementary figure 2: Epistatic interaction for gall number in the Bala x Azucena mapping population. The mean number of galls four weeks after inoculation with *M. graminicola* for all six combinations of allelic combinations of markers in order of RG409 (chromosome 3), RG650 (chromosome 7) and a1245y (chromosome 11) where A is Azucena and B is Bala. Bar is standard error. Bars that do not share the same letter above are different by Tukey's test at $P < 0.05$. Note this a further analysis of data first reported in Shrestha et al., (2007). A three way ANOVA with these markers as factors explains 30% of the variation for gall numbers, and significant interactions are detected between RG409 and a1245y ($P = 0.001$) and between RG650 and a1245y ($P < 0.001$).
